# Supplementary material for: Exploring finite temperature properties of materials with quantum computers
Source: Sci Rep. 2023 Feb 3;13:1986. doi: 10.1038/s41598-023-28317-5 (PMC9898567; doi:10.1038/s41598-023-28317-5)
Supplement: Supplementary file 1 — Supplementary Information. [file 41598_2023_28317_MOESM1_ESM.pdf]

Exploring Finite Temperature Properties of Materials with Quantum Computers
Supplementary Information

Connor Powers*,[1,2] Lindsay Bassman Oftelie,[1] Daan Camps,[1] and Wibe A. de Jong[1]

[1] *Lawrence Berkeley National Lab, Berkeley, CA, USA*

[2] *University of Maryland, College Park, MD, USA*

* *cdpowers@umd.edu*

I. HAAR-RANDOM BEHAVIOR OF RANDOM CIRCUIT STATES

A useful characteristic of a random state $|\psi_R\rangle$ is its entropy H:

$$H_{\psi_R} = -\sum_{k=1}^{2^N} p_k \ln(p_k) \tag{S1}$$

where $p_k \equiv |\langle k|\psi_R\rangle|^2 = |c_k|^2$ [1].

In a true Haar-random state, p_k values follow the Porter-Thomas distribution, which can be shown to lead to $H_{Haar} = \ln(2^N) - 1 + \gamma$ where $\gamma \approx 0.577$ is Euler's constant [2].

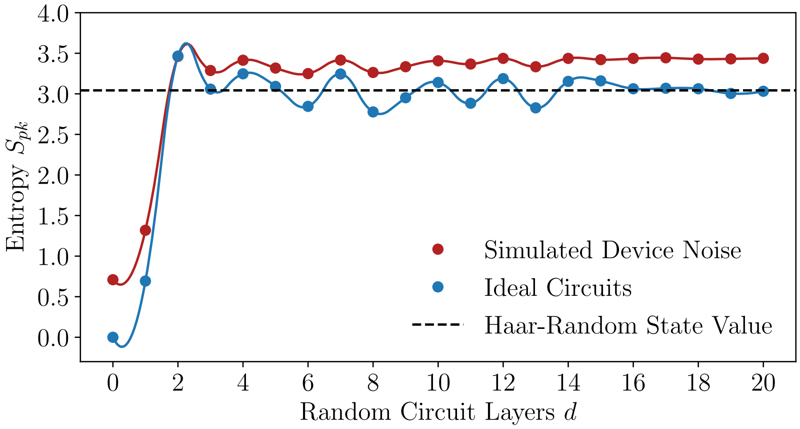

Supplementary Figure S1. Entropy in 5-qubit random states prepared by random circuits of increasing depth d with and without simulated device noise mimicking the IBM "Quito" quantum computer. A B-spline of degree 2 is fit to the data for readability. The entropy of ideal circuits approaches the value expected in a 5-qubit Haar-random state at $d \leq 10$, in line with the findings of Ref. [1], while the simulated device noise causes the entropy to approach a slightly higher value.

As shown in Fig. S1, the entropy of the states generated by this random circuit structure approaches what is characteristic of Haar-random states in relatively few layers when circuits are ideally simulated. The two-qubit gates can be either CZ or CNOT gates, but since CZ sets have shown to converge upon Haar-random values slightly faster than CNOT sets [1], results presented here utilize CZ gates. We also illustrate the effects of simulated device noise; the entropy of the N-qubit random state generated with noisy circuits converges to a value slightly higher than what would be expected of an N-qubit Haar-random state. This is not anticipated to meaningfully affect resulting thermal value estimations.

II. DILATED OPERATOR FIDELITY AND PROBABILITY BEHAVIOR

As mentioned in the main text, the performance of the dilated operator approach to approximating $\hat{Q}$ exhibits complex dependencies on the parameter ϵ. The main performance metrics considered are the fidelity of transformation and the probability of a successful transformation occurring, defined as F and P_0 in the main text respectively, so it is useful to examine how these properties change with ϵ. As an example task, we will seek to determine the thermal energy of a 3-site Heisenberg model with $J_x = 0.5$, $J_y = 1.25$, $J_z = 2.0$, and $h_x = 1.0$ at inverse temperature $\beta = 0.5$.

Fig. S2 shows the thermal energy $\langle H \rangle_{\beta,H}^{TPQ}$ averaged over $R = 100$ independent TPQ states realized using the dilated operator approach for varying ϵ, along with the probability of success P_0, transformation fidelity F, and the true ensemble thermal energy $\langle H \rangle_{\beta,H}^{ens}$.

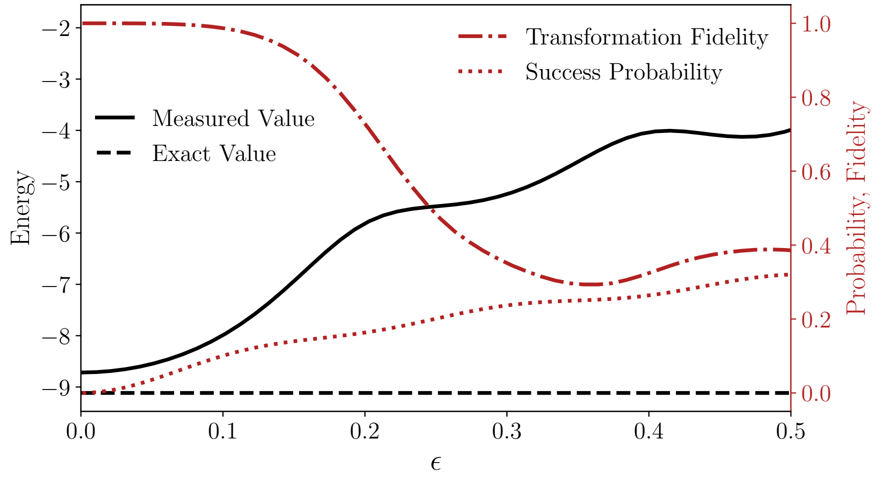

Supplementary Figure S2. Average measured energy, probability of transformation success P_0, and transformation fidelity F of $R = 100$ TPQ states realized via the dilated operator approach for varying ϵ at $N = 5$, $J_x = 0.5$, $J_y = 1.25$, $J_z = 2.0$, $h_x = 1.0$, and $\beta = 0.5$. The ensemble value is shown for reference.

As seen in Fig. S2, the transformation fidelity is highest at low ϵ, while this is where the probability of success is lowest. Therefore, one must pick a value of ϵ such that the transformation fidelity is high enough to recover a good enough approximation of the observable of interest, while making sure the probability of success is large enough to be practical. This threshold will depend on how many shots are feasible to run in a particular research setting.

III. TPQ STATE ERROR TRENDS

Fig. S3a demonstrates observed error trends with increasing system size N, averaged over results from $R = 100$ distinct TPQ states, for a 1D Heisenberg model at inverse temperature $\beta = 0.5$. We define the average squared error as $D(H)^2 = \overline{(\langle \hat{H} \rangle_{TPQ} - \langle \hat{H} \rangle_{ens})^2}$ in alignment with the TPQ state formalism laid out in Ref. [3]. Results are shown from TPQ states formed from random circuits with $d = 2$, when the approximation to a Haar-random state is expected to be poor, and $d = 50$, when this approximation is expected to be close. In the latter case, the average squared error is shown to generally trend down as system size increases, but this trend is not observed when the TPQ states are formed from poor approximations to Haar-random states. This aligns with expectations from Ref. [3]. For low N, the error from utilizing a single TPQ state may become large. Fig. S3b demonstrates that averaging over the results from multiple TPQ states can reduce such error. In this example, the observable of interest is the thermal energy as a function of inverse temperature β, and the simulated system is a 6-spin Heisenberg model.

[1] J. Richter and A. Pal, Simulating hydrodynamics on noisy intermediate-scale quantum devices with random circuits, Physical Review Letters **126**, 10.1103/physrevlett.126.230501 (2021).
[2] S. Boixo, S. V. Isakov, V. N. Smelyanskiy, R. Babbush, N. Ding, Z. Jiang, M. J. Bremner, J. M. Martinis, and H. Neven, Characterizing quantum supremacy in near-term devices, Nature Physics **14**, 595–600 (2018).
[3] S. Sugiura and A. Shimizu, Canonical thermal pure quantum state, Physical Review Letters **111**, 10.1103/physrevlett.111.010401 (2013).

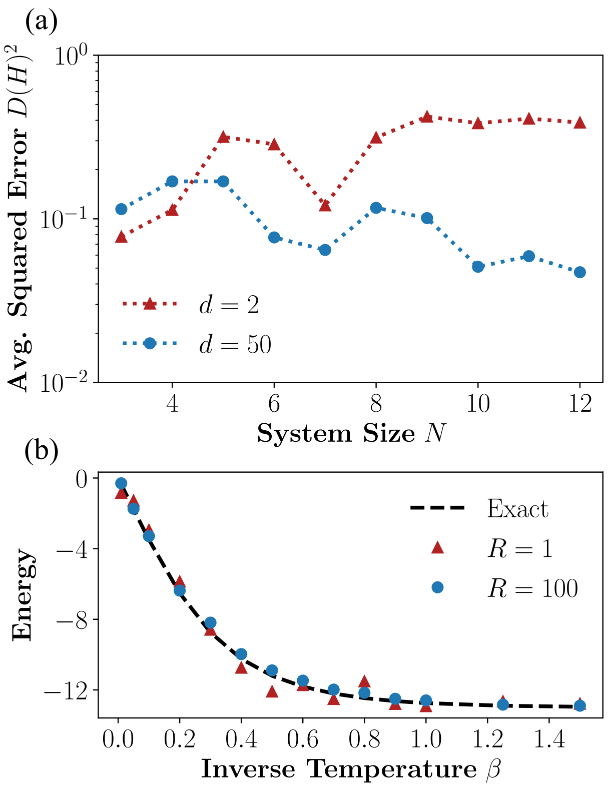

Supplementary Figure S3. (a) Squared error of thermal energy calculated from TPQ states with $d = 2$ and $d = 50$ as a function of system size N for a 1D Heisenberg model at inverse temperature $\beta = 0.5$. Results are averaged over 100 TPQ state realizations. (b) Comparing thermal energies of a 6-spin Heisenberg model as a function of inverse temperature β calculated from a single TPQ state and 100 TPQ states (R=1 and R=100 respectively). Exact values are shown for comparison.
